# Complex clinical manifestations and new insights in RNA sequencing of children with diabetes and *WFS1* variants

**DOI:** 10.3389/fendo.2023.1066320

**Published:** 2023-03-07

**Authors:** Yu Ding, Zhe Li, Qianwen Zhang, Niu Li, Guoying Chang, Yirou Wang, Xin Li, Juan Li, Qun Li, Ru-en Yao, Xin Li, Xiumin Wang

**Affiliations:** ^1^ Department of Endocrinology and Metabolism, Shanghai Children’s Medical Center, Shanghai Jiao Tong University School of Medicine, Shanghai, China; ^2^ CAS Key Laboratory of Computational Biology, Shanghai Institute of Nutrition and Health, University of Chinese Academy of Sciences, Chinese Academy of Sciences, Shanghai, China; ^3^ Department of Medical Genetics and Molecular Diagnostic Laboratory, Shanghai Children’s Medical Center, Shanghai Jiao Tong University School of Medicine, Shanghai, China

**Keywords:** diabetes mellitus, RNA-seq, genetic variation, dilated cardiomyopathy, encephalatrophy, paediatrics

## Abstract

**Background:**

*WFS1*-related disorders involve a wide range of clinical phenotypes, including diabetes mellitus and neurodegeneration. Inheritance patterns of pathogenic variants of this gene can be autosomal recessive or dominant, and differences in penetrance present challenges for accurate diagnosis and genetic counselling.

**Methods:**

Three probands and one elder brother from three families were systematically evaluated and the clinical data of other family members were collected from the medical history. Whole-exome sequencing was performed on the probands, and RNA sequencing was performed on four patients, their parents with *WFS1* variants, and four gender- and age-matched children with type 1 diabetes mellitus.

**Results:**

There were six patients with diabetes. Dilated cardiomyopathy, a rare manifestation of *WFS1*-related disease, was identified in one patient, along with MRI findings of brain atrophy at age 7 years and 3 months, the earliest age of discovery we know of. Whole-exome sequencing revealed five pathogenic or likely pathogenic variants in the *WFS1* gene, including c.1348dupC (p.His450Profs*93), c.1381A>C (p.Thr461pro), c.1329C>G (p.Ser443Arg), c.2081delA (p.Glu694Glyfs*16), c.1350-1356delinsGCA (p.His450Glnfs*26), of which 3 variants (c.1348dupC, c.2081delA, c.1350-1356delinsGCA) were novel that have not been previously reported. The differentially expressed genes were mainly associated with immune-related pathways according to the Gene Ontology enrichment analysis of the RNA sequencing data. The exon 1 region of *HLA-DRB1* in two patients was not transcribed, while the transcription of the region in their parents was normal.

**Conclusion:**

This study emphasizes the clinical and genetic heterogeneity in patients, even in the same family with *WFS1* variants. MRI evaluation of the brain should be considered when *WFS1*-related disorder is first diagnosed.

## 1 Introduction

The *WFS1* gene is located on 4p16.1, spans more than 33.4 kb of genomic DNA and consists of 8 exons. Wolframin 1, a protein encoded by the *WFS1* gene, consists of 890 amino acids. It is a complete endoglycosidase-H sensitive membrane glycoprotein, located in endoplasmic reticulum (ER) and widely expressed in all tissues of the body (1, 2). Wolframin 1 lacks homology with other known proteins and their exact function is not yet elucidated. However, the defects of Wolframin 1 are considered to cause ER stress, damage the cell cycle process, and affect calcium homeostasis (3). According to the Human Gene Mutation Database Professional, most of pathogenic variants of *WFS1* gene are distributed in the coding region, and no variant hotspot has been found.

The pathogenic variation of *WFS1* is inherited in an autosomal recessive or dominant manner. Autosomal recessive inheritance (double allele) often leads to severe Wolfram syndrome 1 (MIM 222300). This syndrome may also be caused by other specific situations, such as uniparental disomy of chromosome 4 (4). One of the typical clinical features of Wolfram syndrome 1 is non-autoimmune insulin-dependent diabetes, with other manifestations such as diabetes insipidus, optic atrophy, and deafness. Autosomal dominant inheritance (heterozygous variation) can lead to Wolfram syndrome like disease (MIM 614296) and most have mild diabetes, independent of insulin treatment (5, 6). Heterozygous variation of *WFS1* can also lead to isolated non-insulin-dependent diabetes (MIM 125853), cataracts (MIM 116400), and deafness associated with pathogenic mutation (automatic dominant 6/14/38) (MIM 600965). The pathogenic variation of *WFS1* is closely related to the occurrence and development of diabetes. We report the clinical and genetic characteristics of six patients with diabetes with *WFS1* variation from three families and further explored their complex clinical phenotypes to deepen the understanding of the disease caused by gene variation.

The clinical phenotype of *WFS1* variation was widely heterogeneous, even within the same family in our clinical cohort. The cause of this wide genetic heterogeneity is unknown and whether it is related to the abnormality of the noncoding region and transcriptome level is worth studying. Of the Mendelian disease-related genes, 70.6% are expressed in the peripheral blood, which greatly improves the feasibility of peripheral blood transcriptome sequencing to study genetic related diseases (7). In 2020, the diagnosis of de Lange syndrome (neurodysplasia related syndrome) was confirmed by sequencing the transcriptome of peripheral blood B lymphocytes (8). In our study, the differential expression data of peripheral blood transcriptome in patients and their parents were analysed for the first time to preliminarily explore the causes for the difference in diabetes phenotype caused by variation of *WFS1*, to improve the accuracy of diagnosis and recognition of this disease.

## 2 Methods

### 2.1 Patients

From January 2018 to November 2021, three children (probands) from three different families, who were hospitalized in our hospital due to diabetes and suspected of monogenic associated syndrome were enrolled in this study. Three probands and the elder brother of one proband were systematically evaluated, including the endocrine and metabolic, urinary, nervous, and cardiovascular systems, and ophthalmology and hearing. The clinical data of other family members were collected. The genetic detection and analysis were performed on probands and thirteen other members in family 1 which was initially described in the previous clinical cohort (9), one proband and four relatives in family 2, and the third proband and parents in family 3. Four gender- and age-matched children with type 1 diabetes mellitus (T1DM) from different families were recruited as a control group and RNA sequencing was performed on their blood samples. This study was approved by the ethics committee of Shanghai Children’s Medical Centre. Written informed consent was obtained from each family. All procedures were conducted in accordance with the principles of the Declaration of Helsinki.

### 2.2 DNA sequencing and sequence analysis

Whole-exome sequencing was performed on three patients (the probands) as previously described (10). The patient in family 2 was simultaneously sequenced for mitochondrial DNA. Briefly, DNA was extracted from patients’ peripheral blood and was then sheared to create fragments of 150–200 bp. Sequencing library was prepared using the SureSelect XT Human All Exon V6 kit (Agilent Technologies, Santa Clara, CA, USA), and sequencing was performed on the Illumina (San Diego, CA, USA) NovaSeq 6000 System. After base calling, quality assessment, and alignment of the sequence reads to the reference human genome (Human 37.3; SNP135), all single nucleotide variants and indels were saved as a VCF format file, which was then uploaded to the Ingenuity^®^ Variant Analysis™ (Ingenuity Systems, Redwood City, CA, USA) for filtering and annotating. The *WFS1* variants identified by whole-exome sequencing were validated by Sanger sequencing using the ABI 3700 sequencer (Applied Biosystems, Foster City, CA, USA) in indicated patients, their parents, and other relatives. The pathogenicity of variation was classified according to the guidelines (11) of the American Academy of Medical Genetics and Genomics, and the variation was divided into benign, likely benign, uncertain significance, likely pathogenic, and pathogenic, which were further improved by the clingen sequence variant interpretation working group (https://www.clinicalgenome.org/Working-groups/Sequence-Variant-expression/).

### 2.3 RNA sequencing and data processing

RNA sequencing was performed in four patients, their parents with *WFS1* variants, and four gender- and age-matched children with T1DM. Whole-blood samples were collected and shipped in Paxgene RNA tubes for processing. At least 1.0 μg of RNA was used for further processing. Isolated total RNA was analysed on an Agilent Bioanalyzer 2100 for RNA integrity number quality check. Globin mRNA was removed before cDNA library construction. All RNA-seq library construction and sequencing steps were performed by Novogene (https://en.novogene.com/). Paired-end 150 bp sequencing was performed on Illumina NovaSeq 6000 instruments. Reads were aligned to the reference human genome (hg38) with STAR v.2.7.1a (12). We used an hg38 genome reference and gencode v.26 for annotation (https://www.gencodegenes.org/human/release_26.html). Principal component analysis (PCA) on gene expression was performed on the basis of TPM (transcript per million) values calculated with the software RNA-Seq by Expectation-Maximization v1.3.1 (13). Differential expression analysis was performed using the DESeq2 R package 1.26.0 (14) and were determined at an adjusted p-value threshold of 0.05. Gene Ontology enrichment analysis of those genes and gene set enrichment analysis of ER stress related genes were performed using the ‘clusterProfiler’ R package 3.12.0 (15). We used Portcullis v1.2.4 (16) for the quantification of junction reads. Sashimi plot of RNA splicing was drawn with ggsashimi v0.5.1 (17).

## 3 Results

### 3.1 Clinical characteristics: description of the patients

**Figure 1:**
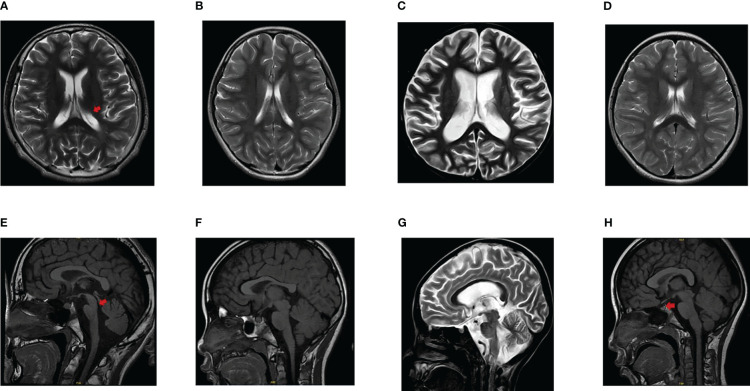
Findings of brain MRI in patients. **(A, E)** The ventricular system was enlarged (arrowhead in A), the sulci and cisterns were widened, the brain stem morphology was poor (arrowhead in E), and lacked a clear high signal indicating the neurohypophysis in patient 1. **(B, F)** The MRI finding presented as an abnormally small neurohypophysis without encephalatrophy in patient 2. **(C, G)** Atrophic changes of the brain was found and lacked a clear high signal indicating the neurohypophysis in patient 5. **(D, H)** No signs of brain atrophy and a normal high signal of neurohypophysis (arrowhead in H) were displayed in patient 6.

A total of six patients with diabetes were found in three families (**Table 1**). Patient 1 and 2 were siblings. Patient 1 was diagnosed at the age of 14. The initial diagnosis was insulin-dependent diabetes, congenital meningocele, neurogenic bladder, amblyopia, and optic atrophy. During the follow-up, hearing impairment occurred and signs of encephalatrophy were found by MRI (**Figures 1A, E**). Patient 2 was diagnosed at the age of 4 years and 9 months, initially diagnosed with T1DM; MRI results showed that the neurohypophysis was abnormally small (**Figures 1B, F**). During the follow-up, she was found to have optic atrophy at 7 years and 2 months of age. The 53-year-old aunt (father’s sister) was diagnosed with high blood glucose levels, which remained elevated for 1 year with a random blood glucose level of more than 10 mmol/L. The doctor at the local hospital diagnosed her with type 2 diabetes and did not provide special treatment. Patient 4 (aunt’s son) had similar clinical manifestations as patient 1. Patient 5, aged 7 years and 3 months, was initially diagnosed with T1DM, neurogenic bladder, optic atrophy, dilated cardiomyopathy, severe anaemia, cardiac insufficiency, and brain atrophy (**Figures 1C, G**). Patient 6 was diagnosed with cataracts due to blurred vision. Routine examination before operation found that blood glucose was increased and no abnormality was found in brain MRI (**Figures 1D, H**). The patients’ adrenal cortex, thyroid, liver, and kidney functions have been normal since follow-up. Patients 5 and 6 were the only children in the family without a family history of diabetes, eye disease, and hearing impairment. The parents of all the patients were non-consanguineous.

**Table 1:** Clinical phenotypic characteristics of patients.

Clinical features	Family 1 (age at diagnosis)				Family 2 (age at diagnosis)	Family 3 (age at diagnosis)
	P1 (14 years old)	P2 (4 years and 9 months old)	P3 (53 years old)	P4 (19 years old)	P5 (7 years and 3 months old)	P6 (6 years and 7 months old)
Gender	M	F	F	M	M	F
BMI (kg/m^2^)	15.62	13.47	27.34	18.51	13.42	15.08
Diabetes	Insulin dependence (6 years old)	Insulin dependence (4 years and 9 months)	Noninsulin dependence (68 years old)	Insulin dependence (2 years old)	Insulin dependence (5 years old)	Insulin dependence (6 years and 7 months old)
Optic atrophy	+ (6 years old)	+ (7 years and 2 months old)	–	+ (15 years old)	+ (6 years old)	–
Cataract	–	–	–	–	–	+ (6 years and 6 months old)
Hearing impairment	+ (15 years and 8 months old)	–	–	–	–	–
Neurogenic bladder	+ (6 years old)	–	–	+ (14 years old)	+ (6 years old)	–
Cardiomyopathy	–	–	–	–	+ (7 years and 3 months old)	–
Meningocele	+ (birth)	–	–	–	–	–
Cerebral atrophy	+ (15 years and 8 months old)	–	NA	NA	+ (7 years and 3 months old)	–

M, Male. F, Female. “+”, Symptom had appeared. “-”, Symptom did not appear. NA, Data not obtained. P1–P6, Patients 1–6.

### 3.2 Identification and *in silico* analysis of the *WFS1* variants in patients

**Figure 2:**
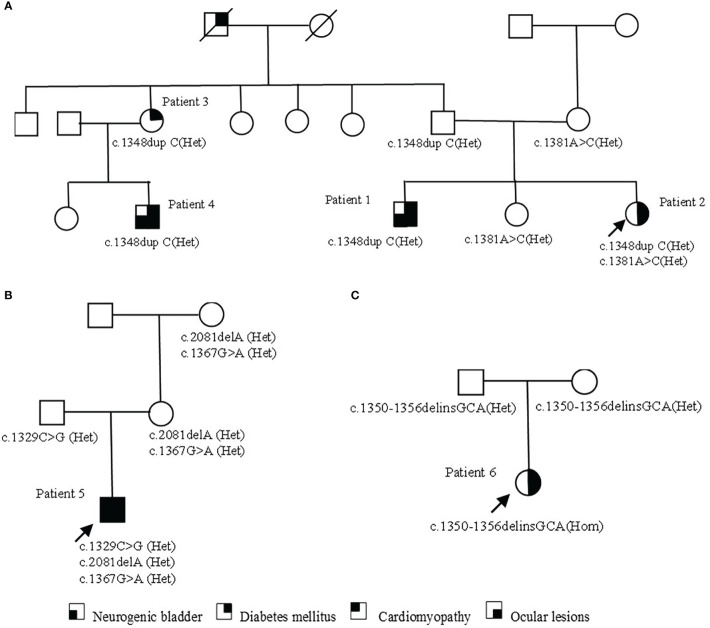
Pedigrees of families with *WFS1* variants. **(A)** The pedigree of family 1. Patient 1 and cousin (Patient 4) carry the same pathogenic variation and have similar clinical symptoms. Patient 3 only shows non-insulin-dependent diabetes; patient 2 carries two heterozygous variants, with a light clinical phenotype. **(B)** The pedigree of family 2. In addition to typical diabetes, optic atrophy, and neurogenic bladder, patient 5 also had a rare cardiomyopathy phenotype. Family members carrying the variant were asymptomatic. **(C)** The pedigree of family 3. The parents of patient 6 were heterozygous carriers and had no clinical symptoms at present.

A total of six *WFS1* variants were found in three families. The c.1367G>A (p.Arg456His) detected in family 2 had a relatively high carrying rate in the population and belonged to the variant without clear meaning. The remaining five variants were determined as pathogenic or likely pathogenic variants according to the American Academy of Medical Genetics and Genomics variation classification standard and their characteristics are listed in **Table 2**. The variant of *WFS1* (c.1348dupC (het), p.His450Profs*93) was detected in family 1. Two patients (Patient 1 and Patient 2), their father, their father’s sister (Patient 3), and their aunt’s son (Patient 4) carried the same heterozygous variation. In addition, patient 2, their mother, and their sister carried c.1381A>C (het) (p.Thr461Pro). No *WFS1* variation was found in other family members. Patient 5 in family 2, his mother, and grandmother carried c.2081delA(p.Glu694Glyfs*16) heterozygous variants. Patient 5 and his grandfather carried the c.1329C>G (p.Ser443Arg) heterozygous variant. The results of mitochondrial DNA sequencing of patient 5 were normal. Patient 6 carries a homozygous variant of c.1350-1356delinsGCA (p.Gis450Glnfs*26), which came from their parents respectively. The distribution of variation in families is shown in **Figure 2**. Three variants (c.1348dupC, c.2081delA, c.1350-1356delinsGCA) were not included in known public databases (i.e., gnomAD, Human Gene Mutation Database, and ClinVar) and were not reported in previous cases, suggesting they were novel.

**Table 2:** Genotype characteristics.

Family ID	Variant	Protein effect	Origin	Variant classification
1	c.1348dupC (Het) c.1381A>C (Het)	p.His450Profs*93 p.Thr461Pro	M/F	P LP
2	c.1329C>G (Het) c.2081delA (Het)	p.Ser443Arg p.Glu694Glyfs*16	M/F	LP LP
3	c.1350-1356delinsGCA (Hom)	p.His450Glnfs*26	M/F	P

Het, heterozygous. Hom, homozygous. M, male. F, female. P, pathogenic. LP, likely pathogenic.

**Figure 3:**
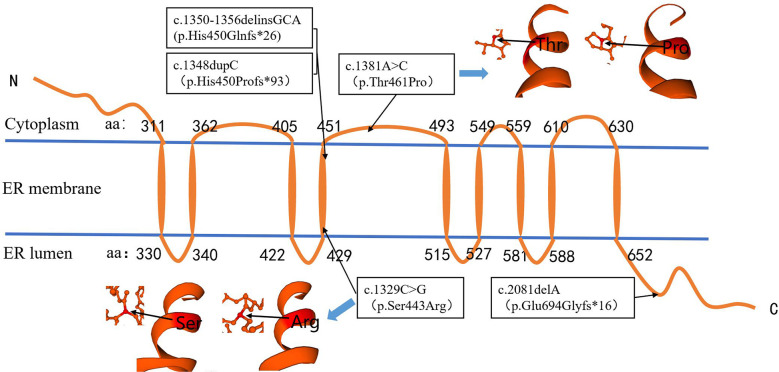
Localization of variation site (P/LP) in protein region and prediction of protein structure in amino acid residue region. The amino acids encoded by c.1348, c.1329, and c.1350–1356 were located in the fourth transmembrane region of WFS1 protein. The amino acids encoded by c.1381 were located in the non-transmembrane region of the cytoplasm. The amino acid encoded by c. 2081 was located in the COOH terminal domain of the endoplasmic reticulum. The c.1381A>C and c.1329C>G variations did not affect the formation of the α-spiral structure.

Among the five sites, two missense variants and three nonsense variants, which could form truncated proteins, were found. The amino acids encoded by c.1350-1356delinsGCA (p.His450Glnfs*26), c.1348dupC (p.His450profs*93), and c.1329C>G (p.Ser443Arg) were located in the fourth transmembrane region of WFS1 protein. The amino acids encoded by c. 1381A>C(p.Thr461Pro) were located in the non-transmembrane region of cytoplasm. The amino acid encoded by c.2081delA (p.Glu694Glyfs*16) was located in the COOH terminal domain of the ER (**Figure 3**), which may have affected the protein function. The online software SWISS-MODEL predicted that the protein structure of the amino acid residues encoded by c.1381A>C(p.Thr461Pro) and c.1329C>G(p.Ser443Arg) variants can form an α-spiral structure, but the local subspace structure was abnormal (**Figure 3**).

### 3.3 Differential gene expression between the patient group and that of parents carrying *WFS1* variants

**Figure 4:**
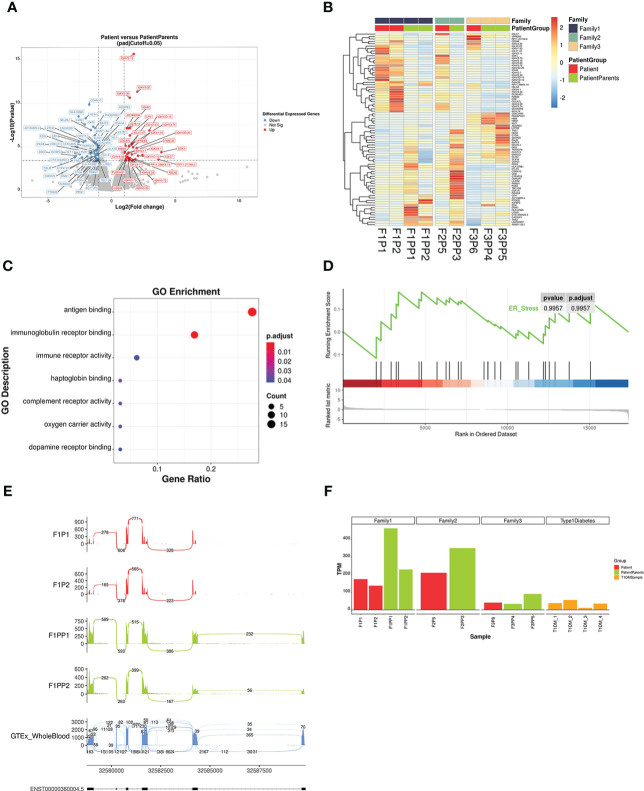
Analysis results of differentially expressed genes and splicing abnormalities by RNA sequencing. **(A)** Volcano plot showing gene expression differences between patients and their parents. Differentially expressed genes with an adjusted *p <*0.05 and a fold-change FC ≥ |2| are depicted in red (up-regulated) and blue (down-regulated). **(B)** Heatmap of all differentially expressed genes. Gene expression levels were quantified by transcript per million and scaled by gene. Red indicates higher expression level while blue indicates lower. **(C)** Gene Ontology (GO) enrichment scatter plot of differential expressed genes. The significance of GO term enrichment is represented by adjusted *p*-value and mapped to the scatter plot by point colour, while point size indicates the number of candidate genes annotated with a GO term. **(D)** Gene Set Enrichment Analysis (GSEA) showed that ER stress related genes exhibited little differential expression. **(E)** Sashimi plot of *HLA-DRB1* gene of family 1 patient and their parents. Exon 1 of *HLA-DRB1* were not transcribed in patients (Patient 1 and Patient 2), compared with their parents as normal transcripts. The last sashimi plot shows the average level of junction reads from 755 GTEx whole blood samples. **(F)** The expression level of *HLA-DRB1* in different samples. The expression level of *HLA-DRB1* was lower in other patients with *WFS1* variants and T1DM, compared to the parent group without diabetes.

Differential gene expression analysis revealed that there were 80 candidate differentially expressed genes in the patient group (P), which contained 47 down-regulated genes and 33 up-regulated genes (**Figures 4A, B**). According to the Gene Ontology enrichment analysis, the differentially expressed genes were mainly enriched to biological processes related to immune function like antigen binding (**Figure 4C**). There was no significant difference in the expression of *WFS1* between the two groups (adjusted *p >*0.05). Gene set enrichment analysis showed that differentially expressed genes were not significantly enriched in ER stress related genes (**Figure 4D**).

### 3.4 Splicing data analysis of RNA transcripts

We found that *HLA-DRB1* in the patient group from family 1 had abnormal splicing based on the splicing data analysis of RNA transcripts. There were six exons in *HLA-DRB1* and the exon 1 region of two patients (Patient 1 and Patient 2) was not transcribed, while the transcription of the region of their parents was normal. Compared with the average reads of 755 whole blood samples from healthy normal humans in GTEx (genetic tissue expression) datasets (18), the junction reads in the front segment of the transcripts in the patient group were relatively low (**Figure 4E**). After quantification of junction read counts with Portcullis, we normalized the junction read counts between exon 1 and exon 2 with the total number of junction read counts at *HLA-DRB1*. The normalized ratio was then transformed to the Z-score. We found that the Z-score of the patient group from family 1 was -1.24, which is relatively low among all GTEx whole blood samples. Differences in *HLA-DRB1* expression were analysed in two other families and in the T1DM group. The expression level of the patient group was lower than that of the parent group, and higher than that of the T1DM group (**Figure 4F**). However, the expression of this gene fluctuated greatly in the peripheral blood according to 755 GTEx whole blood samples (18), with a median value of TPM 89.18, a minimum value of 1.47, and a maximum value of 1147. *HLA-DRB1* was previously reported by GWAS to be associated with asthma (19), rheumatoid arthritis (20) and systemic lupus erythematosus (21), implying its potential role in immune system modulation and it could thus be a candidate modifier of the disease.

## 4 Discussion

Phenotypic penetrance was present in family 1, but the possibility that other alleles that had not been identified might be present could not be ruled out. *WFS1* variants can cause different clinical phenotypes through different inheritance patterns and different degrees of clinical symptoms can also appear in the same inheritance pattern (22, 23). The clinical phenotype was complex and lacked the correlation of gene variation on protein function. In the same family, *WFS1* variants can also lead to different clinical phenotypes, and the presence of autosomal dominant and recessive *WFS1*-related disorders has been described in the same family (24). The lack of genotype-phenotype correlations for *WFS1* variants was also further supported by observations in the probands and their family members in our study.

Reports of *WFS1*-related cardiomyopathy are rare. An OMIM description of the phenotype of Wolfram syndrome 1 (MIM: 222300) proposed a possible clinical phenotype of cardiomyopathy, but no relevant reports were found by searching the literature with the keywords including cardiomyopathy and *WFS1*. Previously reported cardiac abnormalities were mainly structural defects (25, 26). However, in our research group, it was found that Patient 5 developed dilated cardiomyopathy and cardiac insufficiency one year after the diagnosis of diabetes. After 2 months of symptomatic treatment such as with cardiac diuresis and vasodilators, cardiac function improved, and the ventricle became smaller. Based on the clinical presentation of patient 5, differentiation from mitochondrial disease was required. Mitochondrial diseases are a group of heterogeneous multisystem diseases caused by mutations in nuclear or mitochondrial DNA, which can lead to abnormal myocardial structure and function, accompanied by heart failure which worsen dramatically during metabolic crisis (27). No abnormal mutation was found in the patient’s mitochondrial DNA by sequencing, which could preliminarily rule out the disease. Current research suggests that the *WFS1* gene is associated with mitochondrial dysfunction. After silencing the *WFS1* gene in human embryonic kidney cells, mitochondrial dysfunction and up-regulation of related signalling pathway genes lead to cell destruction and degeneration (28). Therefore, the cardiomyopathy of patient 5 may be related to mitochondrial dysfunction caused by *WFS1*. Some irritants such as infection and poor blood sugar control may aggravate the cardiomyopathy and cause clinical manifestations of acute cardiac insufficiency. However, the relationships between *WFS1*, mitochondrial function, and cardiomyopathy need to be further studied. In clinical work, attention should be paid to strengthening the follow-up of cardiac diseases in these patients and regular electrocardiogram and echocardiography examinations will help in early detection and prevent the rapid progression of the disease.

*WFS1*-associated disorders can present as progressive neurodegenerative conditions such as cerebellar ataxia, brainstem dysfunction, peripheral neuropathy, and epilepsy. Cerebral and cerebellar atrophy can even precede the first clinical neurologic signs (29). Limited data is currently available regarding encephalatrophy. The imaging changes of encephalatrophy were found by MRI in patients aged 16–56 (30, 31). The MRI findings of patient 5 showed encephalatrophy at the age of 7 years and 3 months, which is the earliest age of discovery to the best of our knowledge.

The *WFS1* gene encodes a transmembrane protein located in the ER. Current studies have found that missense changes in the terminal domain of COOH can cause sensorineural hearing impairment (32, 33) and Wolfram-like lesions (5, 6). In this study, there was a patient with a variation in this region, which was consistent with previous reports. The four pathogenic variation (P) and likely pathogenic variation sites in this study were all located in exon 8, which was similar to the studies of other populations. Exon 8 is thus a hot spot mutation region (34, 35).

The expression data of *WFS1* transcriptome obtained by RNA sequencing showed that there was no significant difference in the expression of *WFS1* in peripheral blood between the patient and the parent group; we thus speculated that the variation of these sites might affect the spatial structure of WFS1 protein. The variant p.Trp314Arg of WFS1 was found in patients with type 2 diabetes. Patient fibroblasts were analysed using western blotting and immunostaining technology. The results showed that this variation did not change the level of WFS1 protein and subcellular localization, but the variation of *WFS1* (p.Trp314Arg) in HEK293T cell line showed a decrease in the ability to inhibit the ER stress response (23), which further confirmed that variations in *WFS1* may affect protein function without affecting its expression. The natural WFS1 protein is a tetramer structure composed of homologous WFS1 monomers. The tetramer composed of mutant and wild-type monomers may be structurally unsound or have incomplete functionality, leading to the occurrence of disease through a dominant negative mechanism (2), which may be related to the pathogenesis of some patients in the same family.

According to the RNA sequencing results in this study, compared with the parent group, the differentially expressed genes were enriched in the biological processes of antigen binding and immunoglobulin receptor binding. In the temporal lobe of mice with *Wfs1* deleted, gene expression analysis detected the upregulation of growth hormone transcripts and revealed the activation of growth hormone pathways (36). In pancreatic islets of *Wfs1*-deficient mice, RNA sequencing showed that *Wfs1* deficiency significantly influenced the pathways related to tissue morphology, endocrine system development and function, and molecular transport network (37). The gene expression profile of the hypothalamus in *Wfs1* mutant mice indicated a reduction in G protein signalling, which was significantly similar to the profiles of other biological functions (38). Differences in RNA sequencing results may be related to differences in species and sequenced tissues. Peripheral blood is mainly composed of lymphocytes and granulocytes, which may be one of the reasons for more differential expression enrichment in the immune response pathway. However, the possibility of immune system dysfunction caused by *WFS1* gene mutation cannot be ruled out. Currently, most studies of *WFS1* focus on ER stress. Excessive ER stress can affect insulin signalling, insulin biosynthesis, and β cell function, resulting in a reduction of insulin synthesis and sensitivity (39). However, differences in down-regulation or up-regulation of ER stress marker gene transcriptome levels were not statistically significant between the patient and parent group; this is consistent with the RNA sequencing results of pancreatic cells in a *WFS1* mutant mouse model (37). This may be explained by the fact that gene transcription and expression may not always correspond to the protein level; alternatively, *WFS1* may have had less effect on peripheral blood ER stress markers due to its specific expression in tissue and cells.

*HLA* encodes the major histocompatibility complex. Three subregions on DP, DQ, and DR determine the type II major histocompatibility complex molecules of human leukocytes; they play an important role in the mutual recognition of cells that produce immune responses. Genetic polymorphism of *HLA-DRB1* is associated with the occurrence of T1DM. For example, the DRB1*03:01 allele can increase the susceptibility to T1DM in children (40). The first exon of *HLA-DRB1* cannot be transcribed due to abnormal splicing, which may also affect the occurrence and development of diabetes. Previous studies focused more on the risk analysis of *HLA* gene polymorphisms and T1DM, and no report of transcriptome expression data of these loci had been reported. Our study found that the expression of the *HLA-DRB1* transcriptome in the patient group was higher than that in the parent group without diabetes. In addition, the expression of *HLA-DRB1* seemed to be lower in patients with T1DM. However, the expression range of *HLA-DRB1* in peripheral blood of normal people fluctuated greatly. Whether the abnormal expression or splicing of *HLA-DRB1* was related to diabetes was not clear but deserves further attention and experimental investigation.

In conclusion, the clinical phenotypes of diseases caused by *WFS1* variants are complex and accompanied by penetrance insufficiency, which increases the difficulty of diagnosis and genetic counselling of these diseases. For children with insulin-dependent diabetes and optic atrophy, *WFS1* pathogenic variants need to be further excluded; exome sequencing is helpful for the diagnosis of this disease. In these patients, although the incidence of cardiomyopathy is rare, it may still occur, and timely evaluation of cardiomyopathy should be improved during follow-up. Brain atrophy can occur in early childhood and attention should be paid to the evaluation of brain MRIs when the disease is first diagnosed. Peripheral blood RNA sequencing results of patients and carriers suggest that *WFS1* may affect immune-related pathways and the transcriptional expression of *HLA-DRB1* may be related to the pathogenesis of diabetes. However, due to the small sample size, the implications for the occurrence of the disease and the pathogenic mechanism are limited and further studies are needed in the future.

## Data availability statement

The datasets presented in this study can be found in online repositories. The names of the repository/repositories and accession number(s) can be found below: NGDC database (https://ngdc.cncb.ac.cn/), accession number PRJCA012415.

## Ethics statement

The studies involving human participants were reviewed and approved by the ethics committee of Shanghai Children’s Medical Centre. Written informed consent to participate in this study was provided by the participants’ legal guardian/next of kin. Written informed consent was obtained from the minor(s)’ legal guardian/next of kin for the publication of any potentially identifiable images or data included in this article.

## Author contributions

XW and XL (11th author) (co-corresponding authors) designed the study experiments. YD, GC, YW, XL (7th author), JL, and QL were responsible for recruiting patients and collecting clinical features. QZ, ZL, NL, and R-EY were responsible for the sequencing work. YD and ZL drafted the manuscript, tables, and figures. All authors contributed to the article and approved the submitted version.
